# Discontinuity in the Subjective Experience of Self Among People with Mild-To-Moderate Dementia Is Associated with Poorer Psychological Health: Findings from the IDEAL Cohort

**DOI:** 10.3233/JAD-200407

**Published:** 2020-09-01

**Authors:** Linda Clare, Anthony Martyr, Robin G. Morris, Lynette J. Tippett

**Affiliations:** a REACH: The Centre for Research in Ageing and Cognitive Health, College of Medicine and Health, University of Exeter, St Luke’s Campus, Exeter, UK; b Department of Psychology, Institute of Psychiatry, Psychology and Neuroscience, King’s College London, London, UK; c School of Psychology, The University of Auckland, Auckland, New Zealand; d Centre for Brain Research, The University of Auckland, Auckland, New Zealand; e Brain Research New Zealand, Auckland, New Zealand

**Keywords:** Epistemological self, identity, ontological self, quality of life, satisfaction with life, self-persistence

## Abstract

**Background:**

The onset and progression of dementia can result in changes in the subjective experience of self, impacting on psychological health.

**Objective:**

We aimed to explore the extent to which people with mild-to-moderate dementia experience discontinuity in the subjective experience of self, and the factors associated with this experience for people with dementia and their family caregivers.

**Methods:**

We used data from the baseline assessment of the IDEAL cohort. Discontinuity in the subjective experience of self was assessed by asking participants about their agreement with the statement ‘I feel I am the same person that I have always been’. Participants were divided into those who did and did not experience discontinuity, and the two groups were compared in terms of demographic and disease-related characteristics, psychological well-being, measures of ‘living well’, and caregiver stress.

**Results:**

Responses to the continuity question were available for 1,465 participants with dementia, of whom 312 (21%) reported experiencing discontinuity. The discontinuity group experienced significantly poorer psychological well-being and had significantly lower scores on measures of ‘living well’. There was no clear association with demographic or disease-related characteristics, but some indication of increased caregiver stress.

**Conclusion:**

A significant proportion of people with mild-to-moderate dementia describe experiencing discontinuity in the subjective sense of self, and this is associated with poorer psychological health and reduced ability to ‘live well’ with the condition. Sensitively asking individuals with dementia about the subjective experience of self may offer a simple means of identifying individuals who are at increased risk of poor well-being.

## INTRODUCTION

Good psychological health crucially underpins positive evaluations of quality of life, well-being, and satisfaction with life among people with mild-to-moderate dementia [1]. There are many facets to psychological health, including positive self-evaluations and a positive outlook on life, all reflecting subjective personal experience. Hence, the subjective experience of self and personal identity potentially offers a unifying construct that may help to link these facets together. The onset and progression of dementia might result in changes in the experience of self and identity, and impact on psychological health.

Self and identity are difficult concepts to define precisely, and many different approaches have been taken to their measurement, often reflecting different underlying concepts. Models building on the basic distinction between consciousness (where attention is directed outwards) and self-awareness (where attention is directed inwards) offer potential frameworks for understanding the complex phenomenon of self [2]. For example, Neisser’s model [3] proposes five interrelated components of self: ecological, interpersonal, extended, private, and conceptual. Focusing specifically on self and identity among people with dementia, Sabat’s tripartite model [4, 5] considers three components: personal identity, beliefs and attributes, and the personae presented in social interactions. Developing dementia has been considered as a threat to selfhood and identity [6] due to the resulting biographical disruption, which is especially evident in young-onset dementia [7, 8], and the impact on roles and relationships [9], as well as the characteristic impairments in cognition, especially memory, which could crucially affect the experience of self.

Recent work has attempted to reconcile conceptual frameworks and provide clear avenues for empirical investigation. Klein [10] focuses on the distinction between the epistemological or conceptual self (the ‘me-self’) and the ontological self (the ‘I-self’); the former captures the content of, or mental representation of, the self and the latter the phenomenological experience of selfhood. The epistemological or conceptual self is composed of multiple types of self-knowledge that underpin our sense of identity, which can be operationalized and objectively measured. These include, for example, knowledge of one’s personality traits or past life history [3, 11]. The ontological self is the subjective experience of self, which can be described but which is not easy to measure or quantify [10] and which has been examined directly in only a few research studies [12]. Prebble and colleagues [12] add a temporal dimension to this formulation, distinguishing between the present self and the temporally-extended self. Various mental processes enable individuals to integrate diverse aspects of and information relating to the self and experience a coherent sense of identity at any given time, termed ‘synchronic unity’. This sense of continuity extends over time; complex mental processes enable individuals to experience continuity over time, termed ‘diachronic unity’, in both the subjective sense of self (phenomenological continuity) and the self-concept or mental representation of self (semantic continuity). Lifespan developmental research provides consistent evidence that diachronic unity increases with age [13–15]. Typically, people feel that, as they age, they remain the same person, despite experiencing many changes and developments during the course of their lives. In a study examining this phenomenon in healthy individuals aged 85 or over [16], almost all of these older participants considered themselves essentially the same person as they had been in their 20s.

As mental processes are crucially involved, the onset of cognitive impairment may challenge a sense of continuity of self. Episodic and semantic autobiographical memory underpin the conceptual self through maintenance of self-knowledge structures and the ability to generate life story narratives. Episodic memories also contribute to a sense of the persistence of self over time through the ability to relive the past and imagine the future (mental time travel) [12, 15]. Impairments in episodic memory or in semantic autobiographical memory could therefore be expected to undermine both phenomenological and semantic continuity [12]. In general, however, semantic continuity appears relatively robust in the face of cognitive impairment; this can be explained by the observation that the epistemological self is underpinned by multiple, functionally-independent sources of self-knowledge [10], so that impairment or attenuation in some areas is insufficient to affect the overall experience. With regard to dementia, a comprehensive review [9] concluded that the epistemological self is largely maintained in people with mild-to-moderate dementia. Although aspects of episodic and autobiographical memory are affected, resulting for example in less temporal coherence within generated life narratives [17], there are few differences in measures of the epistemological self between people with mild-to-moderate dementia and age-matched healthy controls [18], little change in measures of the epistemological self over a 20 month follow-up period [19] and no linear relationship between decline in cognitive functioning and deterioration in the epistemological self over time [20].

Even though the epistemological self remains relatively unaffected, phenomenological continuity in the ontological self can be affected by the onset of cognitive impairment, and this in turn can be linked to poorer well-being [10]. Evidence about the phenomenological experience of self in people with dementia is limited. Qualitative research indicates that while many individuals feel they are still the same person despite the challenges of living with dementia and the losses involved, others feel that dementia has affected them and their lives to the extent that they are not the same person as before [21]. There has been one in-depth investigation of diachronic unity in a small sample of 14 people with mild-to-moderate Alzheimer’s disease [17], using self-persistence and life-story interviews. The self-persistence interview began with the question ‘Do you feel that you are the same person now as you were when you were in your 20s?’ taken from a previous study of diachronic unity among older people [16]; responses from the participants with Alzheimer’s disease were equally divided between ‘yes’ and ‘no’. However, this question invites comparison with a much earlier life-stage rather than with the pre-dementia experience of self. Furthermore, that study was not designed to examine associations with psychological well-being. The phenomenon of discontinuity among people with dementia has not been explored in large-scale studies, so we do not know how frequent this experience of disruption is, or the extent to which it is associated with psychological well-being for people with dementia and their family caregivers.

In this study we aimed to explore the extent to which people with mild-to-moderate dementia experience a sense of phenomenological discontinuity, the way in which the experience of those who feel they are ‘not the same person’ differs from those who retain a continuous sense of self, and the factors that are associated with disruption to the ontological self for both people with dementia and family caregivers. We hypothesized that the subjective psychological experience of discontinuity: 1) would be more frequent among those with more awareness or acknowledgement of their condition or specific difficulties; 2) would not be clearly linked to demographic or dementia-related characteristics; and 3) would be related to poorer psychological well-being, indicated by poorer scores on a range of psychological indicators as well as measures of ability to ‘live well’ with the condition. If so, it might be a useful predictor of declining ability to ‘live well’ with the condition and a means of identifying individuals who would benefit particularly from additional support in adjusting to life with dementia or targeted interventions to address specific needs.

## METHODS

### Design

We analyzed data from the baseline assessment wave (Time 1, T1) of the Improving the experience of Dementia and Enhancing Active Life (IDEAL) longitudinal cohort study; for protocols see [22, 23]. The analyses were based on v.4 of the IDEAL T1 dataset. IDEAL was approved by Wales Research Ethics Committee 5 (reference 13/WA/0405) and the Ethics Committee of the School of Psychology, Bangor University (reference 2014 11684). IDEAL is registered with the UK Clinical Research Network (UKCRN), number 16593.

### Participants

IDEAL participants were recruited through UK National Health Service memory services and other specialist clinics and via the online Join Dementia Research portal between July 2014 and August 2016. Inclusion criteria were a clinical diagnosis of dementia of any type, in the mild-to-moderate stage as indicated by a Mini-Mental State Examination (MMSE) [24] score of 15 or above, and living in the community at the time of recruitment. Exclusion criteria were inability to provide informed consent, other terminal illness, and any known risk for home visits to pose a risk to researchers. The IDEAL cohort at T1 comprised 1,547 individuals with dementia, of whom 1,283 had a care partner (family member or close friend) also participating. Participants were visited by a researcher on 3 occasions to complete the T1 assessment; people with dementia completed the questionnaires in an interview with the researcher, while caregivers were given their questionnaires to complete by themselves while the researcher was interviewing the person with dementia.

### Measures

Continuity of the ontological self was assessed with the single item ‘I am still the same person as I have always been’, using 5 response options from strongly agree to strongly disagree (0 = strongly disagree, 1 = disagree, 2 = neutral, 3 = agree, 4 = strongly agree). The question was embedded in the second of the three interviews conducted at T1, which focused on psychological health, adjustment to the condition, and well-being.

Awareness, or acknowledgement of the condition or specific difficulties, was assessed with the 9-item screening scale from the Representations and Adjustment to Dementia Index (RADIX) [25]. This was included to allow an exploration of any possible association between extent of acknowledgement of difficulties and sense of discontinuity in the ontological self.

We recorded age, sex, educational qualifications, socio-economic status calculated on the basis of Office for National Statistics [26] classifications, living situation, care partner involvement (whether there was a participating care partner, and if so whether this was a spouse/partner or other relative/friend), dementia diagnosis, and time elapsed since the diagnosis was made. Social network size was assessed with the 6-item Lubben Social Network Scale [27]. The number of co-morbid health conditions present alongside dementia was assessed with the Charlson Comorbidity Index [28, 29] and the age-adjusted score calculated.

Cognition was assessed with the Addenbrooke’s Cognitive Examination-III [30]. Functional ability was assessed with the Dependence Scale [31] and the modified 11-item Functional Activities Questionnaire [32, 33], rated by caregivers where available. Caregivers completed the Neuropsychiatric Inventory Questionnaire [34, 35], yielding scores for number of symptoms observed, severity and resulting distress. Caregiver stress was assessed with the Relative Stress Scale [36].

Positive psychological indicators explored were self-acceptance assessed with the 7-item Self-Acceptance sub-scale of the Ryff Scales of Psychological Well-Being [37] as described in the English Longitudinal Study of Ageing [38], self-esteem assessed with the Rosenberg Self-Esteem Scale [39], self-efficacy assessed with the Generalized Self-Efficacy Scale [40], attitudes towards own ageing assessed with the Attitude Toward Own Aging sub-scale of the Philadelphia Geriatric Center Morale Scale [41], and optimism assessed with the six non-filler items from the Life Orientation Test-Revised [42]. Negative psychological indicators explored were loneliness assessed with the short version of the De Jong Gierveld Loneliness Scale [43] and depression assessed with the 10-item Geriatric Depression Scale [44].

Ability to ‘live well’ with dementia was assessed with the Quality of Life in Alzheimer’s Disease Scale [45], World Health Organization-Five Well-Being Index [46], and Satisfaction with Life Scale [47].

### Statistical analysis

Responses to the continuity question were recoded to form a binary variable. Strongly agree, agree, and neutral responses were combined into a ‘continuity’ category and strongly disagree and disagree responses were combined into a ‘discontinuity’ category.

Analysis of variance and chi-squared tests were used to compare the two groups (discontinuity versus continuity) on demographic characteristics and other variables. Because the large sample size could result in small differences being statistically significant, we calculated eta-squared (*η*^2^) effect sizes for significant comparisons, which were interpreted as follows: small = 0.01; moderate = 0.06; and large = 0.14 [48]. To correct for multiple comparisons, we applied the Holm-Bonferroni correction; only findings that remained significant after correction are reported as such, with associated effect sizes.

## RESULTS

Responses to the continuity question were available for 1,465 participants; the majority agreed (859, 59%) or strongly agreed (199, 14%) that they were still the same person, 95 (6%) were neutral, 282 (19%) disagreed, and 30 (2%) strongly disagreed. Recoding this to a binary variable, 1,153 (79%) agreed or were neutral (continuity group) while 312 (21%) disagreed (discontinuity group). Thus, about 1 in 5 of the sample described an experience of discontinuity in the ontological self.

On the RADIX screening questions, 103 of the 1,465 participants answered ‘no’ to all 9 items, indicating that they did not acknowledge any specific difficulties or changes related to dementia. Of these, 97 (94%) were in the continuity group and only 6 (6%) were in the discontinuity group. Two of those 6 were considered by the interviewer to have acknowledged some dementia-related difficulties at other points during the interview. Thus, the proportion of people in the discontinuity group was lower among those not acknowledging dementia-related changes or difficulties.

Characteristics of participants in the two groups are summarized in Table 1. The average age of participants in the discontinuity group was slightly younger; the difference was statistically significant, but the effect size was negligible. There were no differences between the groups in respect of sex, educational qualifications, socio-economic status, living situation, care partner involvement, social network size, dementia sub-type, time since diagnosis, or age-adjusted number of co-morbid health conditions. Given the lack of differences between the groups we did not control for age or other characteristics in subsequent analyses.

**Table 1:** Comparison of participant characteristics for the discontinuity and continuity groups

Characteristic		Discontinuity (*n* = 312)	Continuity (*n* = 1153)	Comparison	*p*	*η*^2^
Age	Mean	74.72	76.60	F(1,1463) = 12.02	**0.001**	0.008
	SD	9.07	8.35
	N	312	1153
Sex	Male	188	638	*χ*^2^(1) = 2.42	0.120	0.041
	Female	124	515
Educational qualifications	No qualifications	70	325	*χ*^2^(3) = 5.77	0.123	0.064
	School leaving cert age 16	50	206
	School leaving cert age 18	114	379
	University	69	218
	Missing	9	25
Socioeconomic status	I Professional	31	93	*χ*^2^(6) = 10.00	0.124	0.085
	II Managerial and technical	122	375
	III-NM Skilled non-manual	55	228
	III-M Skilled manual	50	248
	IV Unskilled	27	109
	V Unskilled	6	26
	Armed forces	2	17
	Not applicable/missing	19	57
Living situation	Alone	51	215	*χ*^2^(2) = 1.82	0.403	0.035
	With spouse/partner	247	867
	With others	14	65
	Unknown	0	6
Care partner participation	Spouse/partner	214	780	*χ*^2^(2) = 0.70	0.705	0.022
	Other relative/friend	44	184
	No care partner participating	54	189
Lubben Social Network Scale score	Mean	14.22	15.28	F(1,1396) = 6.80	0.009	0.005
	SD	6.06	6.19
	N	294	1104
Dementia diagnosis	Alzheimer’s (AD)	166	647	*χ*^2^(6) = 9.34	0.155	0.080
	Vascular (VaD)	31	128
	Mixed AD and VaD	62	249
	Frontotemporal	12	40
	Parkinson’s disease	13	30
	Lewy body	16	33
	Unspecified/Other	12	26
Time since diagnosis	<1 year	157	618	*χ*^2^(3) = 1.48	0.687	0.033
	1–2 years	96	330
	3–5 years	32	110
	6 + years	3	17
	missing	24	78
Charlson Comorbidity Index age-adjusted score	Mean	7.03	6.98	F(1,1372) = 0.10	0.752	0.000
	SD	2.12	2.22
	N	295	1079

Note: Bold indicates significant at the 5% level after Holm–Bonferroni correction.

Comparisons of cognitive and functional ability and neuropsychiatric symptoms are shown in Table 2. There were no differences between the two groups in cognitive test scores or in care partner ratings of functional ability and dependence. Caregivers of people in the discontinuity group reported a slightly higher number of neuropsychiatric symptoms; this difference was significant, but the effect size was small. Taken together, these findings generally support the hypothesis that discontinuity is not clearly linked to participant or dementia-related characteristics.

**Table 2:** Comparison of cognition, functional ability, neuropsychiatric symptoms and caregiver stress in the discontinuity and continuity groups

Measure		Discontinuity (*n* = 312)	Continuity (*n* = 1153)	Comparison	*p*	*η*^2^
Addenbrooke’s Cognitive Examination-III score	Mean	69.47	69.68	F(1,1368) = 0.06	0.804	0.000
	SD	13.80	12.89
	N	293	1077
Functional Activities Questionnaire rated by caregiver	Mean	18.25	17.55	F(1,1135) = 1.25	0.264	0.001
	SD	8.11	8.68
	N	239	898
Dependence Scale rated by caregiver	Mean	5.80	5.56	F(1,1143) = 1.72	0.191	0.001
	SD	2.63	2.58
	N	235	910
Neuropsychiatric Inventory Questionnaire number of symptoms reported by caregiver	Mean	4.07	3.42	F(1,1158) = 13.25	**<0.001**	0.011
	SD	2.54	2.43
	N	245	915
Neuropsychiatric Inventory Questionnaire severity of symptoms reported by caregiver	Mean	7.24	6.41	F(1,973) = 5.43	0.020	0.006
	SD	4.87	4.57
	N	217	758
Neuropsychiatric Inventory Questionnaire distress at symptoms reported	Mean	8.66	6.73	F(1,868) = 14.55	**<0.001**	0.016
	SD	7.12	5.95
	N	197	673
Relative Stress Scale	Mean	21.02	18.56	F(1,1148) = 12.06	**0.001**	0.010
	SD	9.86	9.70
	N	239	911

Note: Bold indicates significant at the 5% level after Holm–Bonferroni correction.

Psychological indicators and measures of ‘living well’ are summarized in Table 3. These measures did differentiate between the two groups. The discontinuity group had significantly lower scores for self-acceptance, self-esteem, self-efficacy, attitudes towards own aging, and optimism, with moderate or small-to-moderate effect sizes, and significantly higher scores for loneliness and depression, with small-to-moderate effect sizes. The same pattern was observed for measures of ‘living well’. The discontinuity group had significantly lower scores for quality of life, well-being and satisfaction with life, with small-to-moderate effect sizes. These findings supported the hypothesis that discontinuity would be associated with poorer psychological well-being and reduced ability to ‘live well’ with the condition.

**Table 3:** Comparison of psychological indicators and measures of ‘living well’ in the discontinuity and continuity groups

Measure		Discontinuity (*n* = 312)	Continuity (*n* = 1153)	Comparison	*p*	*η*^2^
Ryff Self-Acceptance Sub-scale (7 item)	Mean	29.31	32.69	F(1,1301) = 75.57	**<0.001**	0.055
	SD	6.67	5.48
	N	277	1026
Rosenberg Self-Esteem Scale	Mean	27.70	29.96	F(1,1347) = 84.86	**<0.001**	0.059
	SD	4.07	3.55
	N	282	1067
Generalized Self-Efficacy Scale	Mean	27.04	29.86	F(1,1365) = 61.40	**<0.001**	0.043
	SD	5.85	5.23
	N	279	1088
Philadelphia Geriatric Center Morale Scale Attitudes toward own Ageing	Mean	1.84	2.70	F(1,1156) = 52.97	**<0.001**	0.044
	SD	1.59	1.64
	N	241	917
Life Orientation Test	Mean	13.85	15.27	F(1,1429) = 40.25	**<0.001**	0.027
	SD	3.82	3.34
	N	302	1129
De Jong-Gierveld Loneliness Scale	Mean	1.87	1.23	F(1,1437) = 44.23	**<0.001**	0.030
	SD	1.72	1.41
	N	301	1138
Geriatric Depression Scale (10 item)	Mean	3.55	2.44	F(1,1317) = 51.37	**<0.001**	0.038
	SD	2.57	2.17
	N	260	1059
Quality of Life in Alzheimer’s Disease	Mean	34.35	37.40	F(1,1336) = 60.36	**<0.001**	0.043
	SD	6.17	5.70
	N	274	1064
World Health Organization-Five Well-Being Index Well-Being Index	Mean	51.55	63.47	F(1,1445) = 84.06	**<0.001**	0.055
	SD	22.48	19.45
	N	303	1144
Satisfaction with Life Scale	Mean	22.92	26.94	F(1,1435) = 113.06	**<0.001**	0.073
	SD	6.72	5.62
	N	306	1131

Note: Bold indicates significant at the 5% level after Holm–Bonferroni correction.

There was some indication that caregivers of people in the discontinuity group fared worse than caregivers of people in the continuity group (see Table 2). They reported significantly greater distress at the neuropsychiatric symptoms they observed and higher levels of caregiver stress, but with small effect sizes.

## DISCUSSION

This is the first large-scale study to explore the experience of phenomenological continuity and discontinuity in the ontological self, and the associations with other variables, among people with mild-to-moderate dementia. Participants in the IDEAL cohort were asked whether they felt they were ‘still the same person’ that they ‘have always been’. The 21% who disagreed with this statement, indicating a sense of discontinuity in the subjective sense of self, experienced significantly poorer psychological well-being across a range of indicators, and had significantly lower scores on measures of ‘living well’ with dementia, than their counterparts who reported retaining a subjective sense of continuity, and almost all showed at least some awareness of the condition or associated changes or difficulties. There was no clear association with demographic or disease-related characteristics, but some indication of increased levels of stress in caregivers.

Discontinuity was less frequent in our sample than the 50% reported in the one previous small-scale study of this phenomenon among people with dementia [17]; discontinuity was reported by half of the participants with Alzheimer’s disease (*n* = 14) and half of the age-matched healthy controls (*n* = 25). Given that the group of people with dementia in this earlier study both included participants with more extensive cognitive impairments (MMSE score of 10 and above) and found that those who provided more sophisticated explanations for their response to the continuity question were more likely to report a subjective sense of discontinuity, one might perhaps have expected to see higher levels of discontinuity in the present sample. However, Troll and Skaff [16] reported that almost all of their ‘oldest old’ participants considered they were essentially the same person as they were in their 20s. In general, research from a lifespan developmental perspective indicates that self-continuity increases over the lifespan, with older people experiencing a well-defined and stable sense of self whereas young adults are still engaged in developing their identity [13]. Therefore, the findings of the present study could be considered more in line with existing evidence.

A possible explanation might be the difference in the wording of the question, as the question used in the earlier study required a direct comparison to a specific earlier life-stage (the 20s) whereas in our study the question was phrased more generally as the ‘same person you have always been’. Asking for a direct comparison with a past life-stage might lead participants to focus more on differences between their past and present selves, whereas the more general wording might be more likely to predispose participants to consider similarities. For people with dementia, the more generally-worded question inviting comparison with the ‘person you have always been’ may be more likely to invite comparison with the pre-dementia self in the relatively recent past, and hence provide a more representative estimate of the extent to which people experience a sense of discontinuity as they develop and live with mild-to-moderate dementia. Further research could explore the issues associated with the question used in our study and the version used by Tippett and colleagues [17] in more detail in order to provide a clearer picture of the phenomenon elicited and allow for comparisons between people with dementia and older people without dementia.

Those individuals who acknowledge and openly discuss their diagnosis of dementia could perhaps be asked more directly about perceived differences to the pre-dementia self. In IDEAL, we phrased the question in general terms because some participants in this large survey might not acknowledge, or be aware of, their diagnosis and/or any associated difficulties. In the present study, 7% of participants responded negatively to a series of screening questions asking about perceived difficulties, symptoms, and changes [25], intended to identify those who did not acknowledge, or who lacked awareness of, the condition. Almost all of these participants also felt themselves to be the ‘same person’ they had always been. An apparent lack of awareness might arise for a number of reasons, with responses influenced by perceptions of socially appropriate answers, psychological mechanisms of avoidance, or neurologically-based anosognosia [49]. However, it seems plausible to expect that participants who perceived no dementia-related changes or difficulties might also be less likely to report a sense of discontinuity, and our findings support this view.

The finding that the subjective sense of discontinuity was associated with poorer psychological health while not reflecting demographic or disease-related differences points to its validity as an indicator of a potentially important aspect of individuals’ subjective psychological experience. Associations were seen in relation to self-evaluative judgements regarding self-acceptance, self-esteem and self-efficacy, and attitudes towards own aging, as well as extending more generally to mood, optimism and loneliness. This in turn was reflected in lower levels of quality of life, well-being and satisfaction with life. While a myriad of factors show small associations with the quality of life of people with dementia [50], statistical modelling of IDEAL cohort data has demonstrated the key role of psychological characteristics and health in shaping individual evaluations of quality of life, well-being and satisfaction with life [51]. As those who report a subjective sense of discontinuity are likely to be experiencing lower psychological well-being and/or more psychological distress, identifying these individuals may offer a relatively simple means of focusing on those who would benefit from additional support.

Caregivers of participants reporting a subjective sense of discontinuity had slightly increased levels of stress. Again, multiple factors may influence the experience of family caregivers [1, 52], and study of reciprocal influences within the caregiving dyad is at a relatively early stage. However, previous actor-partner independence modelling of IDEAL cohort data demonstrated that depression in the person with dementia was associated with reduced quality of life, well-being and satisfaction with life in the care partner [53], while perceived relationship quality had no such effect [54]. Thus, the psychological health of both members of the dyad may be interrelated, with poor psychological health in the person with dementia contributing to greater stress in the carer, and vice versa. Addressing the psychological needs of one member of the dyad may also improve well-being for the other.

Our approach of investigating the experience of continuity or discontinuity in a cohort of people with mild-to-moderate dementia has a number of strengths, including the large sample size, inclusion of people with a range of dementia sub-types and other characteristics, and availability of data from an extensive set of measures. However, there are some limitations that must be considered.

The most important of these, also comprehensively discussed by Tippett and colleagues [17], relates to the way in which respondents understand and interpret the question used to assess discontinuity. Even though these types of question appear to directly address the issue, it is difficult to know whether someone who reports a sense of discontinuity really experiences him- or herself subjectively as a fundamentally different person or whether the response reflects a considered, analytical attempt to engage with the implications that cognitive decline and dementia may hold for sense of self. This reflects the key challenge inherent in attempting to elicit and measure the subjective experience of self as opposed to elements of self-knowledge that can be more objectively operationalized and assessed [10]. Nevertheless, our observation that participants who did not acknowledge dementia-related difficulties were extremely unlikely to report a sense of discontinuity supports the view that the question asked in the present study is relevant and potentially useful in this context. In this analysis we did not consider the possible implications of differing degrees of certainty in responding, as numbers in the ‘strongly disagree’ category in particular were small. We were not able to compare the responses of our participants with dementia to those of other groups and hence cannot determine the extent to which the profile elicited is specific to people living with dementia. Similarly, there might be generational or cultural differences in how people respond to questions about continuity of self, but as our sample, recruited through health services in 29 areas of Great Britain, consisted largely of individuals from a white British background, we were not able to explore this possibility. Further research could compare responses among those with and without dementia and among different groups of people living with dementia, and assess the utility of other methods of exploring this phenomenon. Another issue to acknowledge is that while the question was asked only once at this time-point, subjective perceptions of self might be influenced in the moment by a range of factors and hence responses could potentially change over relatively short periods of time and in different contexts. Finally, the cross-sectional nature of our study allows only for the observation of associations; however, as longitudinal data from the IDEAL cohort become available, we will be able to explore both the predictive value of the experience of discontinuity and the stability of responses over time.

In view of the association with poorer well-being, the question arises as to whether a subjective sense of discontinuity might be reduced or changed through intervention. There is relatively little evidence available regarding the potential of interventions to support self and identity in people with dementia [55]. Supporting identity is sometimes considered either as a specific aim of an intervention or as a possible outcome, but without measuring identity or objectively assessing the impact the intervention has on identity alongside other possible benefits. Examples of such interventions include Selbst-Erhaltungs-Therapie (self-maintenance therapy) [56], use of videos composed of personal photographs [57], and music-based interventions [58]. Only a handful of interventions both explicitly aim to support identity and assess this as an outcome; examples include engagement in activities relevant to personal identity roles [59] and life review or life story work [60, 61]. These interventions focus on aspects of self-knowledge and re-connecting individuals with elements of their past identities. Further research might explore the potential to address issues of self and identity directly through targeted intervention.

While theoretical and methodological questions remain to be answered, our findings have one key practical implication. In busy clinical settings where extensive assessment of psychological well-being is impractical, a sensitive exploration of whether the person is experiencing a sense of discontinuity of self, perhaps by weaving a question like ‘Do you feel as if you are the same person you have always been?’ into the conversation, offers a potential means of identifying individuals who may be at increased risk of poor psychological well-being and who may benefit from additional services or support, both for themselves and for any family member providing care. This should, of course, be done with caution and sensitivity, and only in situations where the topic can be properly considered, any emotional response explored satisfactorily, and any necessary follow-up provided.

### Conclusions

This study represents the first large-scale exploration of discontinuity in subjective sense of self among people with mild-to-moderate dementia. It has shown that a significant proportion of people with mild-to-moderate dementia describe a subjective experience of discontinuity, and that this is associated with poorer psychological health and reduced ability to ‘live well’ with the condition, as well as slightly increased levels of stress in family caregivers. Further investigation may provide greater clarity about the phenomenon and its implications, and the potential for intervention. Meanwhile, sensitively asking individuals with dementia about the subjective experience of self may provide busy clinicians and practitioners with a useful means of identifying individuals who may be at increased risk of poor well-being and who may benefit from additional support.

## Data availability

IDEAL data were deposited with the UK data archive in April 2020 and will be available for access from April 2023. Details of how the data can be accessed after that date can be found here: http://reshare.ukdataservice.ac.uk/854293/.
